# Indoxyl Acetate as a Substrate for Analysis of Lipase Activity

**DOI:** 10.1155/2019/8538340

**Published:** 2019-12-01

**Authors:** Tomas Valek, Adam Kostelnik, Pavla Valkova, Miroslav Pohanka

**Affiliations:** ^1^Faculty of Military Health Science, University of Defense, Trebesska 1575, 50001 Hradec Kralove, Czech Republic; ^2^Faculty of Pharmacy, Charles University, Akademika Heyrovskeho 1203, 50005 Hradec Kralove, Czech Republic

## Abstract

Lipases play a crucial role in metabolism of microbes, fungi, plants, and animals, and in analytical chemistry, they are often used in detection of fats and triglycerides. Determination of lipase activity is also important in toxicology, when lipase activity can be both increased and decreased by organophosphates and other pesticides and in medicine for diagnosis of heart diseases. The standard method for lipase activity determination is based on cleaving ester bonds in lipase buffer containing Tween. Our aim was to find a method with faster and more sensitive response. It is known that acetylcholinesterase belongs to the same group of hydrolases enzymes as lipases and it cleaves indoxyl acetate, so we assume indoxyl acetate could report a similar reaction with lipase. Our method is based on indoxyl acetate as a substrate for lipase, where indoxyl acetate is cleaved by lipase to indoxyl and acetate moiety and blue indigo is created. The method was optimized for different times and amount of enzyme and compared with the standard Tween assay. The calibration curve measured in reaction time 20 minutes with 10 *μ*l of lipase exhibited the best analytical parameters, and it showed Michaelis–Menten response with the Michaelis–Menten constant equal to 8.72 mmol/l. The indoxyl acetate-based method showed faster and more sensitive response than the standard method for lipase activity determination, so it has great potential in biosensor construction and it could be used in industry, medicine, toxicology, and common practice where the activity of lipases is need to be measured.

## 1. Introduction

All lipases belong to triacylglycerol hydrolases (E.C. 3.1.1.3) class of enzymes. Their function is to cleave ester bonds in fats, mostly triglycerides, to glycerol and fatty acids. Lipases are known as serine hydrolases because catalytic center contains serine, histidine, and aspartate or glutamate, depending on the type of organism [1–4]. However, active center of lipases containing serine, aspartate, and histidine with open/close conformation is mainly same for all types of lipases. Lipases occur in many variations with respect to source, and they differ in specific properties of enzyme such as pH, temperature, stability in the presence of metal ions and organic solvents, and substrate specificity. These factors have influence to activity and properties of enzyme [5, 6].

Lipases have important role in metabolism, their activity can be found in animal, plant, fungi and microbe organisms, and they mostly have important function during digestion of fats [7, 8]. They also have an important place in the industry, medical applications, and biotechnology [9–11]. Nowadays, importance of lipases has been growing in the medical sector because admission of an external enzyme can even serve as cancer prevention [12]. It appears that lipoprotein lipases with abnormal function correspond with pathological processes like obesity, Alzheimer disease, atherosclerosis, and dyslipideamia associated with diabetes and infections [13]. Beside that lipases have important role in toxicology, their activity can be both increased and decreased by some toxic compounds including organophosphates, carbamates, and ethanol. [14–16]. Organophosphates and carbamates are used in agriculture, medicine, and industry, and they have been developed by chemical manipulation of extremely toxic nerve gasses. Their well-known effect is based on inhibition of acetylcholine esterase (AChE) which leads to disorders of neurotransmission, but they affect the whole metabolism and biochemical pathways [17–19].

Our intention was to develop new, fast, sensitive, and accurate method for lipase activity assay based on substrate indoxyl acetate (IA). IA is not so well-known as a substrate for lipase activity determination, and it has been applied rarely so far and analytical parameters do not exist or are hardly available. For example, IA was used as a substrate for lipase activity determination in work of Purr and the method was based on test-paper impregnated by IA [20]. Test-paper was proved as easy to handle, but sensitivity and other analytical parameters have been found as highly insufficient, so IA was not further tested as a suitable substrate for lipase activity determination [21]. IA is also one of used substrates for AChE activity detection. Effect of AChE on IA is to cleave IA to indoxyl and acetate moiety, followed by spontaneous blue indigo creation. Coloration of solution is measurable by spectrophotometry at wavelength 620 nm [22, 23].

AChE just as lipases belongs to serine hydrolases group (E.C. 3.1.1.), and its catalytic triad consists of three amino acids, serine, histidine, and glutamate, very similar to catalytic triad of lipases [24–26]. That is why we assumed that IA could be also used as a substrate for determination of lipase activity. Based on previous experiences with IA as a substrate for lipase activity determination, we see the potential in IA as a substrate, so we focused on the improvement of analytical properties of the new IA-based method.

## 2. Materials and Methods

### 2.1. Chemicals

All chemicals were obtained from commercial sources in analytical grade, and no adjustment was done on chemicals before use. Indoxyl acetate (IA), phosphate-buffered saline (pH 7.4), Tween 20, lipase from porcine pancreas (Type VI-S, ≥20,000 units/mg protein, lyophilized powder), tris(hydroxymethyl)aminomethane hydrochloride (Tris-HCl), CaCl_2_, and Tween 20 were bought from Sigma-Aldrich (St. Louis, MO, USA), and absolute ethanol was purchased from Penta (Prague, Czech Republic). Lipase buffer was composed from 20 mmol/l Tris-HCl, 80 mmol/l CaCl_2_, and 1% Tween 20. Temperature of all chemicals was adapted to laboratory conditions, and all experiments were performed under standard ambient temperature and pressure (SATP).

### 2.2. Apparatus

Color change of measured solutions was detected by using the 96-channel spectrophotometer, Tecan Sunrise (Salzburg, Austria). Demineralized water was prepared by reverse osmosis process using device Aqua Osmotic 2 (Aqua Osmotic, Tisnov, Czech Republic). Buffers and other solutions were prepared using the pH meter, CyberScan pH 6000 from Eutech (Landsmeer, The Netherlands).

### 2.3. Standard Method Based on Tween 20

Analysis of Tween 20 as a substrate for lipase was used as standard method for determination of lipolytic activity. Our measurements were inspired by work of Plou and coauthors with minor revisions [27]. We used Tween 20 in concentration range from 0 to 50 mmol/l (0, 6.25, 12.5, 25, and 50 mmol/l) in lipase buffer mixed together with lipase (200 U), and we measured absorbance of solution in time range from 0 to 60 minutes using wavelength 450 nm.

### 2.4. IA-Based Method

**Figure 1:**
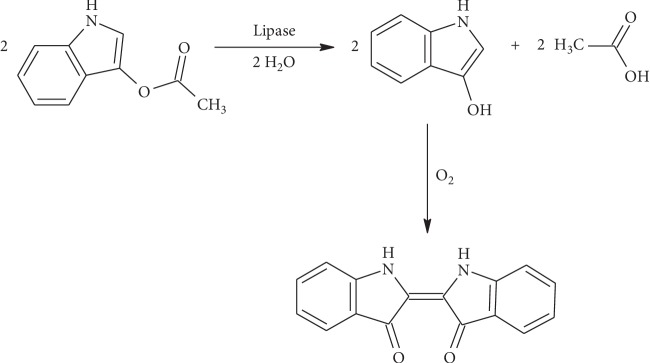
Scheme of IA cleaving by lipase to acetate and indoxyl and spontaneous creation of blue indigo.

To prove IA as a substrate for lipase activity detection, the assay was performed in the concentration range of IA 0–50 mmol/l (0, 6.25, 12.5, 25, and 50 mmol/l). Total volume of solution was 200 *μ*l, IA was added in quantum of 40 *μ*l and lipase in two different amounts 5 *μ*l or 10 *μ*l (100 and 200 U), and residue volume was completed by phosphate buffer. Absorbance of solution was measured in the time range from 0 to 30 minutes using wavelength 620 nm. The principle of reaction based on lipase cleaving IA is shown in Figure 1.

### 2.5. Spontaneous Coloration of IA

IA in concentration range from 0 to 50 mmol/l (0, 6.25, 12.5, 25, and 50 mmol/l) was measured with and without enzyme in time for determination of possible spontaneous coloration of IA. Forty microliters of IA was mixed together with 160 *μ*l of phosphate buffer or with 150 *μ*l of phosphate buffer and 10 *μ*l of lipase. Solutions were measured by using the spectrophotometer with wavelength 620 nm in time range from 0 to 30 minutes.

### 2.6. Docking of IA to Lipase

UCSF Chimera (version 1.11.2; developed by RBVI with support of NIH, University of California, San Francisco [28]) was used for creating 3D images and in silico prediction. Human pancreatic lipase (PDB code: 2PVS [29]) was fetched and prepared for docking using Dock Prep tool. For docking, AutoDock Vina tool was then used, and calculation was run online on Opal server.

### 2.7. Data Processing

All measurements were performed in pentaplicate. Measured data were processed by software Origin 9.1 (OriginLab Corporation, Northampton, Massachusetts, USA). Signal vs. noise equal to three criterions (S/N = 3) was used for LOD calculation. Michaelis–Menten constant (*K*_m_) for lipase and IA or Tween 20 was calculated using nonlinear curve fitting and Hill function with the coefficient of cooperativity *n* = 1.

## 3. Results

### 3.1. Standard Method Based on Tween 20

**Figure 2:**
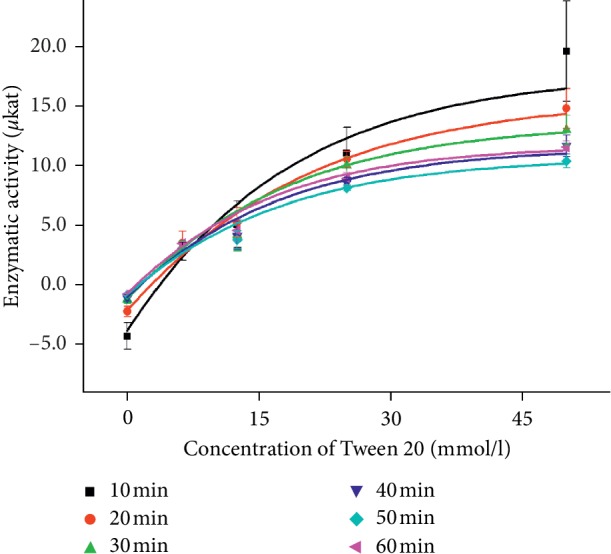
Concentration curves of Tween (0, 6.25, 12.5, 25, and 50 mmol/l) measured with 10 *μ*l of lipase (200 U) by the spectrophotometer at the wavelength 450 nm in the time range of 0–60 minutes. The experiment was performed under SATP. Error bars indicate the standard deviation for *n* = 5.

Increasing absorbance responds to increasing concentration of Tween by Michaelis–Menten behavior, as it is shown in Figure 2 measured by the spectrophotometer at wavelength of 450 nm. Statistic data of curves measured in time are summarized in Table 1. Significantly, the lowest *K*_m_ and limit of detection (LOD) were determined in the curve measured in the reaction time of 60 minutes.

### 3.2. IA-Based Method

**Figure 3:**
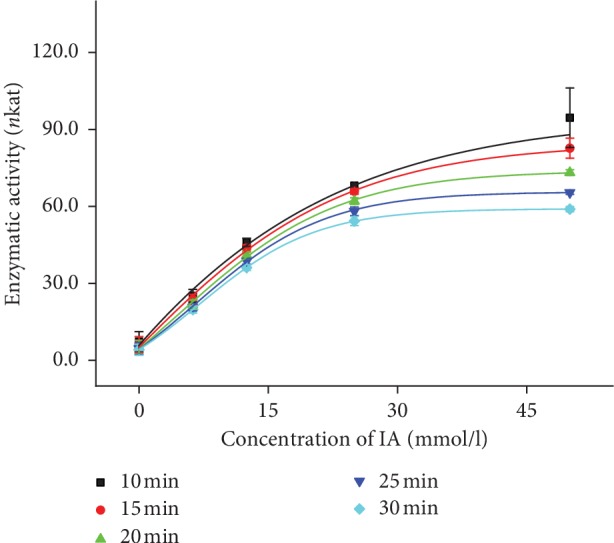
Concentration lines of IA (0, 6.25, 12.5, 25, and 50 mmol/l) measured by the spectrophotometer at 620 nm wavelength in the time range of 0–30 minutes. Curves belong to the volume 5 *μ*l of lipase (100 U). The experiment was performed under SATP. Error bars indicate the standard deviation for *n* = 5.

**Figure 4:**
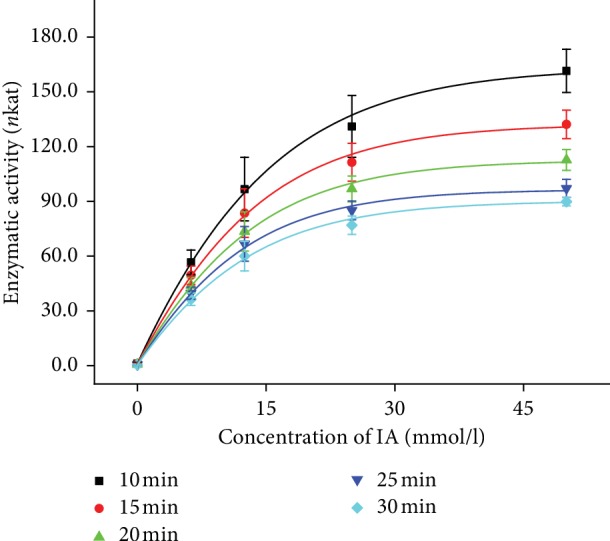
Concentration lines of IA (0, 6.25, 12.5, 25, and 50 mmol/l) measured by the spectrophotometer at 620 nm wavelength in the time range of 0–30 minutes. Curves belong to the volume 10 *μ*l of lipase (200 U). The experiment was performed under SATP. Error bars indicate the standard deviation for *n* = 5.

Due to hydrolytic function of AChE, ability to cleave ester bonds in IA, it was an assumption that lipase would have same process of reaction [23]. IA is used as a substrate for AChE activity determination, so it inspired us to use IA as a substrate for lipase activity determination. During the optimization of the assay, volume of lipase was experimentally determined and 10 *μ*l of lipase (200 U) was set as ideal volume, but concentration range of IA was also measured with volume of lipase 5 *μ*l (100 U). Curves measured with both 5 *μ*l and 10 *μ*l of lipase show Michaelis–Menten dependence (Figures 3 and 4), but the results measured with higher volume of lipase show better Michaelis–Menten dependence. Data of concentration curves for 5 *μ*l and 10 *μ*l of lipase are summarized in Tables 2 and 3. Optimization of time of reaction was also done, and curves for each time are shown in Figures 3 and 4. Because of minimal difference between results calculated from curves measured at 20, 25, and 30 minutes, the suitable time of measurement was set to be 20 minutes as the shortest time of assay. Increasing absorbance corresponds to the increasing concentration of IA, so we assumed IA is possible to be used as a suitable substrate for lipase activity determination.

### 3.3. Spontaneous Coloration of IA

**Figure 5:**
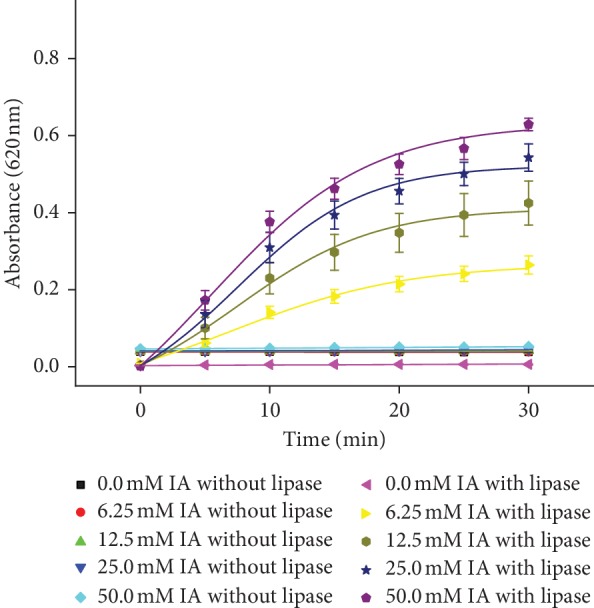
Coloration of IA in the time range of 0–30 minutes for different IA concentrations (0, 6.25, 12.5, 25, and 50 mmol/l) measured with and without lipase. The experiment was performed under SATP. Error bars indicate the standard deviation for *n* = 5.

Increasing absorbance of measured solution could be also the result of spontaneous coloration of IA, so we measured IA with and without enzyme. Results in Figure 5 show no significant changes of absorbance with increasing time for each IA concentration while the enzyme was not used. Using enzyme results showed significant increase in time for each IA concentration beside 0 mmol/l concentration. Since we recorded no spontaneous coloration of IA during assay time, we assume that the presented method is not influenced by the spontaneous coloration of IA.

### 3.4. Docking of IA to Lipase

**Figure 6:**
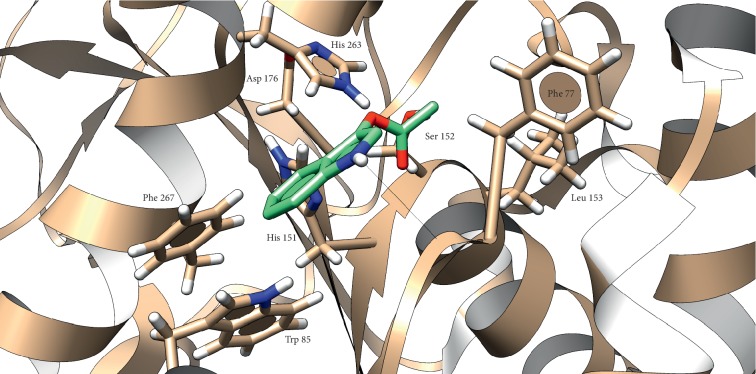
Presumed position of IA in human pancreatic lipase. Atom color: white = H, red = O, blue = N, and grey (in lipase) and green (in IA) = C. Black line represents the hydrogen bond.

There was a hydrogen bond between Leu 78 and hydrogen atom associated with nitrogen in IA molecule (2.28 Å). This interaction is stabilized by *π*-*π* interaction of IA aromatic system with aromatic amino acids in lipase structure (Figure 6). Binding energy predicted for this interaction is −0.5 kcal/mol. Parameters and findings predicted by in silico method are summarized in Table 4.

## 4. Discussion

In comparison of the standard method with Tween 20 and the method using IA, we reached better results in shorter time using IA. The IA-based method shows lower *K*_m_ than standard assay so higher sensitivity and affinity of enzyme to substrate was proved. Increase of enzymatic activity was monitored as a function of concentration at different times. Sensitivity can be improved by the prolongation of time analysis; on the other hand, too long time of analysis is not required in the standard processes. The suitable time of analyses was established in time 20 minutes, because measured analytical parameters are sufficient enough and the method does not respond much better (analytical parameters are not significantly improved) by the prolongation of time. In work of Hernández-García and coauthors [30], different substrates were tested using lipases from two sources *Mucor javanicus* and *Pseudomonas fluorescens*. *K*_m_ values were calculated for used substrates, where the lowest value was proven for palmitate using lipase from *Mucor* and myristate using the lipase from *Pseudomonas*; the values of *K*_m_ were calculated to be 0.95 and 0.57 mmol/l, respectively. They used environment with higher temperature (50–60°C) depending on the source of lipase. These values are lower than ours approximately ten times, but during our measurement, higher temperature was not necessary to be used; on the other hand, we can also assume a decrease in the *K*_m_ value with the increasing temperature for our IA substrate. It is also necessary to mention that, for different substrates, the *K*_m_ values were comparable with our values (laurate *K*_m_ = 11.49 mmol/l for lipase from *Mucor*), which means the affinity of lipase to our substrate is sufficient and comparable with that in other studies. In [31], *p*-nitrophenylphosphate was tested as a substrate for lipase activity determination, and the affinity of enzyme (lipase from *Pseudomonas cepacian*) to substrate was evaluated on base of *K*_m_ value equal to 12 ± 1.6 mmol/l; these results correspond to our measurements. From the available data, we can evaluate smaller substrate molecules (IA, *p*-nitrophenylphosphate) have very similar *K*_m_ values little bit higher than for larger substrate molecules (palmitate and myristate), but mainly the *K*_m_ values depend on the source of lipase. The turnover number for porcine lipase and IA as substrate was also calculated, and it is in the range of 0.087–0.183 (Tables 2 and 3). Similar results were achieved using eugenol (0.67 s^−1^) and fungi lipase [32], triacetin (0.1 and 0.32 s^−1^), and also 4-nitrophenyl acetate (0.36 and 0.44 s^−1^) using lipase from rice [33] and 4-nitrophenyl butyrate (0.125 and 0.158 s^−1^) using lipase genetic mutants of *Staphylococcus xylosus* [34]. For comparison, the turnover number for tributyrin using lipase genetic mutants from *Staphylococcus xylosus* was published to be in range from 450 to 1900 s^−1^, depending on the type mutant [35] and even higher in camel and scorpio models (4333 s^−1^ and 5370 s^−1^ respectively) [36, 37].

Among the *K*_m_ values, IA has more advantages in comparison with standard Tween substrate. The reaction of lipase with IA is colored, so it is visible by naked eye and semiquantitative detection without use of device is possible. This fact could have one very good application. Sometimes, screening for identification of lipase/esterase producing bacteria without previous purification of enzyme is required. The test is performed by cultivation of the specific microbe onto surface of agar plate in Petri dish. Usually, the tributyrin hydrolysis test is used to screen lipase-producing bacteria, and for the analysis of lipase activity, the titrimetric method has to follow the tributyrin test [38–40]. Among tributyrin, triolein is also often used as a substrate for lipase-producing bacteria identification, but addition of fluorescent, for example, rhodamine B is required, and the test is more complicated than the tributyrin test in practice mainly due to inconsistent effect. Tween with addition of Nile blue indicator can be also used for this type of test, but the visible clearance crucial for determination of lipase activity is unclear [39]. IA could be an ideal substrate for the determination of lipase presence in the tested type of microorganism because the color change would occur under bacterial colony in the case of active lipase presence and the color change would be detectable by naked eye without the application of any fluorescent or color indicator. In works of Żądło-Dobrowolska and coauthors, fluorescent probes were designed for detection of lipase, esterase, and protease activities based on the utilization of the carbamate cleavable linkage in a probe structure [41, 42]. Although the fluorescent probe method is highly sophisticated, the probe is stable under aqueous conditions and not susceptible to nonspecific degradation and the IA based method is much simpler and more cost effective. Thus, it is easier to observe blue coloration of agar enriched by IA solution than to perform any other already known test. In the future, addition of IA to agar plate could be tried as given in the following test.

Interaction of IA with pancreatic lipase was studied in silico. As no crystal structure of porcine pancreatic lipase was found in PDB database, we decided to use human pancreatic lipase which is structurally very similar to the porcine one. It was important to use lipase with lid in an open conformation because it simulates active enzyme in the physiological environment. From Figure 5, it should be noted that the orientation of carbonyl moiety in the IA molecule is toward residues Phe 77 and Leu 153, which stabilize the formed intermediate in oxyanion hole during interaction with Ser 152 [29]. This could be taken as clue about proper orientation of IA in the lipase active site; additionally, IA is a very small molecule compared to long chains of fatty acids, so the interaction with hydrophobic amino acids in long cavity enter is not expected. As can be seen from the results, a small amount of energy was released suggesting that lipase has low affinity to IA. Compared with the energy released during cleavage of natural substrates like tripalmitin or tributyrin, the affinity of lipase to IA seems to be low; however, it still can be used as lipase substrate with additional benefit of coloring when it is used for screening of lipase activity of microbial cultures on agar plate [43, 44]. On the other hand, the affinity to substrates varies between types of lipase and used substrate as can be learned from the literature [30, 31]. Despite the theoretical background supporting our findings, it is still only prediction and it should be confirmed by X-ray crystallography.

## 5. Conclusions

Some methods using IA as suitable substrate for lipase activity determination have been already described, but a good sensitivity and other analytical results were not observed. There is no work using IA as a substrate with comparable results to the standard method, and our assay gives a new look on IA as a convenient substrate for lipase activity determination. In our opinion, our IA-based method has a useful potential in analytical chemistry and could be used as a suitable, fast, and easy method for lipase activity detection. During measurements, the standard spectrophotometry method for lipase activity determination was compared with IA-based spectrophotometry method. The improved method with use of IA is unpretentious, fast, and more sensitive. The method is easy and not so difficult for preparation and time duration. During measurements, the *K*_m_ value was lower in the IA-based method than that of the standard method, thus it shows a higher sensitivity of lipase to the substrate. The novel method with IA is suitable for common utilization in the industry, medicine, analytical chemistry, and toxicology for lipase activity determination.

## Figures and Tables

**Table 1:** Statistic data of concentration curves of Tween in time the range of 0–60 min.

Time (min)	*K*_m_ (mmol/l)	*V*_max_ (*μ*kat)	*R*	*k*_cat_ (s^−1^)	LOD (mmol/l)
10	16.4	20.0	0.982	19.2	2.73
20	16.7	20.0	0.997	19.2	1.45
30	13.9	10.0	0.995	9.60	1.25
40	13.1	10.0	0.997	9.60	0.935
50	13.0	10.0	0.998	9.60	0.497
60	12.0	10.0	0.998	9.60	0.380

*K*_m_—Michaelis–Menten constant, *V*_max_—maximum velocity, *R*—correlation coefficient, *k*_cat_—turnover number, LOD—limit of detection. The values belong to the volume 10 *μ*l of lipase (200 U).

**Table 2:** Statistic data of concentration curves of IA in the time range of 0–30 min.

Time (min)	*K*_m_ (mmol/l)	*V*_max_ (*n*kat)	*R*	*k*_cat_ (s^−1^)	LOD (mmol/l)
10	13.6	95.1	0.998	0.183	3.67
15	12.3	84.7	0.999	0.163	3.15
20	11.2	74.0	0.999	0.142	3.00
25	10.4	65.7	0.999	0.126	2.89
30	9.85	59.1	0.999	0.114	2.78

*K*_m_—Michaelis–Menten constant, *V*_max_—maximum velocity, *R*—correlation coefficient, *k*_cat_—turnover number, LOD—limit of detection. The values belong to the volume 5 *μ*l of lipase (100 U).

**Table 3:** Statistic data of concentration curves of IA in the time range of 0–30 min.

Time (min)	*K*_m_ (mmol/l)	*V*_max_ (*n*kat)	*R*	*k*_cat_ (s^−1^)	LOD (mmol/l)
10	9.27	163	0.999	0.157	0.209
15	9.16	133	0.999	0.127	0.122
20	8.72	113	0.999	0.108	0.102
25	8.12	96.5	0.999	0.093	0.100
30	8.49	90.3	0.999	0.087	0.092

*K*_m_—Michaelis–Menten constant, *V*_max_—maximum velocity, *R*—correlation coefficient, *k*_cat_—turnover number, LOD—limit of detection. The values belong to the volume 10 *μ*l of lipase (200 U).

**Table 4:** Facts about IA and human pancreatic lipase interaction predicted by the in silico method.

Parameter	Findings
Predicted binding energy	−2092 J/mol
Predicted interactions	H-bond: Leu 78
	*π*-*π* interaction: Trp 85, His 151, Phe 267

## Data Availability

All data used to support the findings of this study are included within the article.
